# The Effect of Chronic Alcohol on Cognitive Decline: Do Variations in Methodology Impact Study Outcome? An Overview of Research From the Past 5 Years

**DOI:** 10.3389/fnins.2022.836827

**Published:** 2022-03-10

**Authors:** Annai J. Charlton, Christina J. Perry

**Affiliations:** ^1^Florey Institute of Neuroscience and Mental Health, Parkville, VIC, Australia; ^2^Florey Department of Neuroscience and Mental Health, University of Melbourne, Parkville, VIC, Australia; ^3^School of Psychological Sciences, Centre for Emotional Health, Macquarie University, North Ryde, NSW, Australia

**Keywords:** alcohol, alcohol use disorder, cognitive decline, stress, behavior, animal models

## Abstract

Excessive alcohol use is often associated with accelerated cognitive decline, and extensive research using animal models of human alcohol consumption has been conducted into potential mechanisms for this relationship. Within this literature there is considerable variability in the types of models used. For example, alcohol administration style (voluntary/forced), length and schedule of exposure and abstinence period are often substantially different between studies. In this review, we evaluate recent research into alcohol-induced cognitive decline according to methodology of alcohol access, as well as cognitive behavioral task employed. Our aim was to query whether the nature and severity of deficits observed may be impacted by the schedule and type of alcohol administration. We furthermore examined whether there is any apparent relationship between the amount of alcohol consumed and the severity of the deficit, as well as the potential impact of abstinence length, and other factors such as age of administration, and sex of subject. Over the past five years, researchers have overwhelmingly used non-voluntary methods of intake, however deficits are still found where intake is voluntary. Magnitude of intake and type of task seem most closely related to the likelihood of producing a deficit, however even this did not follow a consistent pattern. We highlight the importance of using systematic and clear reporting styles to facilitate consistency across the literature in this regard. We hope that this analysis will provide important insights into how experimental protocols might influence findings, and how different patterns of consumption are more or less likely to produce an addiction-vulnerable cognitive phenotype in animal models.

## Introduction

Accelerated cognitive decline is a feature of alcohol use disorder and can have important implications for treatment retention and relapse propensity (Bates et al., 2002). While some alcohol-induced cognitive deficits are acute and subside with sobriety, others can persist for years into abstinence (Stavro et al., 2013); in severe cases even leading to irreversible dementia (Hoffman et al., 2019). Cognitive domains that appear particularly susceptible to the effects of alcohol are cognitive flexibility, learning and memory, decision-making and inhibition (Noël et al., 2007; Woods et al., 2016). In humans, these are commonly assessed *via* comprehensive test batteries such as Cambridge Neuropsychological Test Automated Battery (CANTAB, Donoghue et al., 2020), or using specific cognitive behavioral tasks such as the Stroop Test (Ioime et al., 2018), Wisconsin Card Sorting Task (Reynolds et al., 2019; Faustino et al., 2021), or Iowa Gambling Task (Reynolds et al., 2019). Importantly, impairments within these cognitive domains are thought to contribute to high relapse rates and poor treatment outcomes (Bates et al., 2002; Trick et al., 2014; Worley et al., 2014). Despite decades of research, however, there is still no targeted treatment for alcohol-induced cognitive decline, and the definitive mechanism remains elusive (Perry, 2016).

Determining the amount of alcohol that may lead to cognitive decline is important for developing safe drinking guidelines. It has been suggested that low to moderate doses of alcohol may have protective effects against dementia (Xu et al., 2017; Rehm et al., 2019), however the reliability of these positive associations has been questioned (Chikritzhs et al., 2015), and findings from other studies do not report the same dose-related protective effects (Hassing, 2018). In fact, a recent review (Brennan et al., 2020) concluded that the sum evidence available was often inconsistent, with high risk of bias arising due to study design limitations (inability to randomize or adequately control, and residual confounds). Unfortunately, such limitations make it unlikely that we can obtain a clear answer regarding dose-dependent effects of alcohol on human behavior and cognition with the current data available. While it is difficult to disentangle the role of factors such as dose and exposure length in human studies, animal models can be a useful way to assess the effect of individual factors and the mechanisms underpinning these effects, in a systematical and controlled way.

In this manuscript, we review recent studies that have used animals to model the effect of chronic alcohol on behavior. The basic model has animals (usually rodents) exposed to alcohol *via* voluntary or non-voluntary means for a protracted period. Following this, cognitive decline is assayed *via* a range of different behavioral tests which we will describe in the following section. While these animal models are useful tools to recapitulate aspects of human alcohol use disorders, and for studying the mechanism underlying alcohol-induced cognitive deficits, within the current literature there is substantial variability in the methods used. This makes it difficult to accurately interpret and compare results from different studies. This review is intended as a tool to compare and contrast current animal models, to identify where alcohol-induced cognitive deficits have, or haven’t been seen, and to assess how methodological variation may impact the nature and severity of these cognitive deficits. We were interested in whether there is a relationship between the severity of the deficit and the amount of alcohol consumed, the schedule of alcohol access and length of abstinence, the age of alcohol exposure, and the sex of subjects. The cognitive functions assessed by different behavioral tasks, and their value and relevance, will also be evaluated. We hope that this analysis will provide important insights into how experimental protocols might influence findings, and into how different patterns of consumption are more or less likely to produce an addiction-vulnerable cognitive phenotype in animal models. Ultimately, our aim is to provide guidance on the value of different animal models of human alcohol use disorder.

To conduct this review, keywords: “alcohol or ethanol,” “cognition,” “cognitive decline,” and “chronic” were searched in the PubMed database, with results limited to the last 5 years. This yielded 292 manuscripts. From here, manuscripts were selected based on abstracts; including only original research that specifically examined the effect of alcohol, and included some type of behavioral test aimed at assessing cognitive performance. Most studies were conducted on rodents, so these were the focus of the review. However, some non-human primate studies were also included. While many studies addressed both behavioral changes and neural mechanisms, this review focused on behavior measuring cognitive performance only. In addition, this review looked at the direct effects of alcohol and did not include additional pathologies, such as Alzheimer’s Disease, thought to be exacerbated by alcohol (Hoffman et al., 2019). Many of the studies cited also investigated potential interventions. However, assessment of interventions and biological mechanisms is beyond the scope of the current review. Furthermore, given the volume of research carried out on this question, we limited our search to the past 5 years. We note the existence of comprehensive reviews that cover earlier literature (Vetreno et al., 2011; Perry, 2016; Staples and Mandyam, 2016).

Within the studies addressed, there was substantial variability across studies in the subject characteristic (species, age, and sex), although most research was in male rodents. While genetic differences between strains no doubt can affect alcohol intake, metabolism, and sensitivity to cognitive deficits, there was insufficient evidence available to explore this in the current review. However we have added information about the genotype tested.

There were a range of methods used for alcohol administration: some were enforced, such as gavage, intraperitoneal (i.p.) injection or vapor inhalation; while others relied on voluntary consumption, such as a two-bottle choice. The details of each of these administration methods are outlined in the section below. In addition to variable administration methods, there were also varied schedules. The length of access/administration varied from a few days to several months. Within this time, access could be multiple times a day, every other day, or a number of iterations in between. This led to variation in intoxication levels, reported as both grams of alcohol per kg of bodyweight, and average blood alcohol concentration (BAC). Following cessation of alcohol, some studies had a very short withdrawal period before commencing behavioral testing, while in others, animals were abstinent for weeks.

The behavioral tests used were often analogs of those used in humans, and ranged from assessing spatial learning and memory, discrimination learning and memory, reversal-learning and set-shifting. Through these tests, patterns emerged. For example, impaired behavioral flexibility appeared robust, while impaired acquisition of a discrimination task (i.e., working memory) was only occasionally observed. Each of these are outlined in more detail through the review. For a more expansive description of different behavioral tests assessing cognitive flexibility, please see Highgate and Schenk (2021).

As this review is intended as a tool to help researchers understand how specifics of experimental protocol might influence study outcomes, we have created a series of tables organized by behavioral task. These contain information regarding age/sex/species/genotype of subjects, method of administration, duration of administration, magnitude of intake, length of abstinence, and whether cognitive deficits were present. Table 1 shows Spatial Learning and Retrieval tasks; Table 2, Working Memory and Discrimination; Table 3, Reversal Learning; and Table 4, Set-shifting.

**TABLE 1:** Spatial learning and retrieval summary from reviewed studies outlining behavior task, effect, methodology and subjects.

Behavior Task	Effect in Acquisition/[Reversal]	Voluntary/Non-Voluntary	Method of Administration	Alcohol exposure (days)	Abstinence before test	Intake (g/kg)	Species: Genotype	Sex: M/F	Age of exposure	References
Barnes Maze	√ [√]	Non-voluntary	Gavage	5	11–13 days	5 g/kg	Rat: Wistar	N/S	Adult – N/S	Gibula-Tarlowska and Kotlinska, 2020
Barnes Maze	× [√]	Non-voluntary	Gavage	5	11–13 days	5 g/kg	Rat: Wistar	M	Adult	Marszalek-Grabska et al., 2018
Barnes Maze	× [√]	Non-voluntary	Gavage	30	3 weeks	5 g/kg	Rat: Sprague Dawley	M	Adolescent	Fernandez and Savage, 2017
Barnes Maze	× [√]	Non-voluntary	Vapor	21–28	5–7 days	N/S- BEC 200 mg/dL	Mouse: C57BL/6J	M	Adult	Varodayan et al., 2018
MWM	√	Non-voluntary	Drinking water	84	N/S	N/S	Rat: Wistar	M	Adult	Pandey et al., 2020
MWM	√	Non-voluntary	Drinking water	84	N/S	N/S	Rat: Wistar	M	Adult	Dwivedi et al., 2018
MWM	√	Non-voluntary	Gavage	56	11 h	10 g/kg	Rat: Sprague Dawley	M	Adult	Chen and Hu, 2017
MWM	√	Non-voluntary	Gavage	14	N/S	N/S	Mouse: Kunming	M + F	Adult	Xing and Zou, 2018
MWM	√	Non-voluntary	Gavage	30	1 day	N/S	Rat: Wistar	M	Adult	Vaghef et al., 2019
MWM	× [√]	Non-voluntary	Gavage	30	25 + days	5 g/kg	Rat: Wistar	M	Adolescent	Vetreno et al., 2020
MWM	M: × [×] F: √ [×]	Non-voluntary	Gavage	4	4.5 days	8.8 g/kg (F), 8.3 g/kg (M)	Rat: Long-Evans	M + F	Adult	Maynard et al., 2018
MWM	×	Non-voluntary	Gavage	1, 5 or 10	3–5 days	5 g/kg	Mouse: C57BL/6J	M	Adult	Wang et al., 2018
MWM	√	Non-voluntary	Gavage	28	24 h	3 g/kg	Rat: Wistar	M	Adolescent	Pamplona-Santos et al., 2019
MWM	√	Non-voluntary	Gavage	4	5 days	11–15 g/kg/day	Rat: Wistar	M	Adult	Cippitelli et al., 2017
MWM	×	Non-Voluntary	IP	5	16 days	4 g/kg/day	Rat: Sprague Dawley	M	Neonate	Swart et al., 2017
MWM	√	Non-voluntary	IP	42	N/S	5 mg/kg	Mouse: C57BL/6N	M	Adult	Ikram et al., 2019
MWM	√	Non-voluntary	Liquid diet	28	none	N/S	Mouse: C57Bl6	M	Adult	Wang et al., 2017
MWM	√ [√]	Non-voluntary	Liquid diet	49	21 days	9 g/kg (adult), 7 g/kg (Aged)	Rat: Sprague Dawley	M	Adult + Aged	Novier et al., 2016
MWM	√	Voluntary	2BC	43	N/S	>15 g/kg	Mouse: Swiss Albino	M	Adult	Rajput et al., 2017
MWM	×	Voluntary	2BC	28	N/S	7–11 g/kg/day	Mouse: Swiss Albino	M	Adult	Pant et al., 2017
MWM	√	Voluntary	4BC	48	N/S	∼20 g/kg	Mouse: C57BL/6	M	Adult	Liu et al., 2019
NOR	√	Non-voluntary	Liquid Diet + Gavage	16	7 h	N/S	Mouse: C57BL/6	F	Adult	King et al., 2020
NOR	√	Voluntary	DID	28	2 weeks	∼7 g/kg/4 h	Mouse: C57BL/6J	M	Adolescent	Rico-Barrio et al., 2019
RAM	√	Non-voluntary	Gavage	5	15 days	1.5 g/kg/day	Rat: Long-Evans	M	Adolescent	Loxton and Canales, 2017
T-maze	√	Non-voluntary	Gavage	4	4 days	5 g/kg	Rat: Long-Evans	M + F	Adult	West et al., 2018
T-maze	√	Non-voluntary	Drinking Water	∼180	none, 1 or 6 weeks	N/S	Mouse: C57BL/6	M	Adult	Dominguez et al., 2016
T-maze	√	Non-voluntary	Vapor + 2BC	37–54	2–3 days	2BC 1.5–2.5 g/kg/2 h	Mouse: C57BL/6	M	Adult	Pradhan et al., 2018
Y-maze	√	Non-voluntary	Drinking water	28	N/S	N/S	Rat: Sprague Dawley	M	Adolescent – N/S	Du et al., 2019
Y-Maze	√	Non-voluntary	IP	42	N/S	5 g/kg	Mouse: C57BL/6N	M	Adult	Ikram et al., 2019
Y-Maze	√	Non-voluntary	N/S	84	N/S	1.5 g/kg/day	Mouse: C57BL/6J	M	Adult	George et al., 2018

*M, male; F, female; MWM, Morris water maze; NOR, novel object recognition; RAM, radial arm maze; Gavage, intragastric or oral gavage; IP, intraperitoneal injection; Vapor, vapor inhalation chamber; Liquid Diet, Lieber DeCarli liquid ethanol diet; Drinking Water, alcohol in drinking water; DID, drinking in the dark; 2BC, two-bottle choice; 4BC, four-bottle choice; Self-Admin, Oral self-administration; N/S, Not explicitly stated; g/kg, grams per kilogram; h, hour; mg/dL, milligrams per deciliter.*

**TABLE 2:** Working memory and discrimination summary from reviewed studies outlining behavior task, effect, methodology and subjects.

Behavior Task	Effect in Acquisition/[Reversal]	Voluntary/Non-Voluntary	Method of Administration	Alcohol exposure (days)	Abstinence before test	Intake (g/kg)	Species: Genotype	Sex: M/F	Age of exposure	References
Discrimination- Olfactory	IP: × [√] Gavage: [x]	Non-voluntary	IP + Gavage	14–42	5–7 days	3–5 g/kg	Rat: Sprague Dawley	M	Adult	Badanich et al., 2016
Discrimination- Olfactory	Adolescent: × [√] Adult: √ [√]	Non-voluntary	Gavage	25	24 h or 3 weeks	5 g/kg	Rat: Sprague Dawley	N/S	Adolescent, Adult + Aged	Fernandez et al., 2017
Discrimination- Operant	× [√]	Non-voluntary	Gavage	4 days	5 days	11–15 g/kg/day	Rat: Wistar	M	Adult	Cippitelli et al., 2017
Discrimination- Operant Touchscreens	× [√]	Voluntary	2BC	180 days	3 days	∼6 g/kg	Rat: Long-Evans	M	Adult	Charlton et al., 2019
NOR	√	Non-voluntary	Gavage	28	24 h	3 g/kg	Rat: Wistar	M	Adolescent	Pamplona-Santos et al., 2019
NOR	√	Non-voluntary	Gavage	7	24 h	3 g/kg	Rat: Wistar	M	Adult	Geiss et al., 2019
NOR	×	Non-voluntary	Gavage	30	3 weeks	5 g/kg	Rat: Sprague Dawley	M	Adolescent	Fernandez and Savage, 2017
NOR	√	Non-voluntary	Gavage	30	108 days	5 g/kg	Rat: Wistar	M	Adolescent	Vetreno et al., 2016
NOR	√	Non-voluntary	IP	5	16 days	4 g/kg/day	Rat: Sprague Dawley	M	Neonate	Swart et al., 2017
NOR	√	Non-voluntary	IP	1	48 h	3 g/kg	Rat, Sprague Dawley	M	Adolescent	Drissi et al., 2020
NOR	√	Non-voluntary	IP	16	24 h or 3 weeks	1.25 g/kg	Mouse: OF1	M	Adolescent + adult	Ledesma et al., 2017
NOR	√	Non-voluntary	IP	24	8 days	3 g/kg/day	Rat: Wistar	M	Adolescent	Sanchez-Marin et al., 2017
NOR	√	Non-voluntary	N/S	84	N/S	1.5 g/kg/day	Mouse: C57BL/6J	M	Adult	George et al., 2018
NOR	√	Non-voluntary	Vapor	28	N/S	N/S	Mouse: C57BL/6J	M	Adult	Nie et al., 2018
NOR	√	Non-voluntary	Vapor + 2BC	37–54	2–3 days	2BC: 1.5–2.5 g/kg/2 h	Mouse: C57BL/6	M	Adult	Pradhan et al., 2018
NOR	√	Voluntary	2BC	43	N/S	>15 g/kg	Mouse: Swiss Albino	M	Adult	Rajput et al., 2017
NOR	×	Voluntary	2BC	28	N/S	7–11 g/kg/day	Mouse: Swiss Albino	M	Adult	Pant et al., 2017
NOR	√	Voluntary	4BC	49–56	<1 week	1.4–2.2 g/kg/24 h	Rat: Wistar	M	Adolescent	Silva-Peña et al., 2019
NOR	√	Voluntary	DID	28	2 weeks	∼7 g/kg/4 h	Mouse: C57BL/6J	M	Adolescent	Rico-Barrio et al., 2019
Object/context mismatch	×	Non-voluntary	Vapor + Drinking Water	29	72 h	0.75–1 g/kg/h	Mouse: C57BL/6J	M	Adult	Rodberg et al., 2017
Passive Avoidance	×	Non-voluntary	N/S	84	N/S	1.5 g/kg/day	Mouse: C57BL/6J	M	Adult	George et al., 2018

*M, male; F, female; MWM, Morris water maze; NOR, novel object recognition; RAM, radial arm maze; Gavage, intragastric or oral gavage; IP, intraperitoneal injection; Vapor, vapor inhalation chamber; Liquid Diet, Lieber DeCarli liquid ethanol diet; Drinking Water, alcohol in drinking water; DID, drinking in the dark; 2BC, two-bottle choice; 4BC, four-bottle choice; Self-Admin, Oral self-administration; N/S, Not explicitly stated; g/kg, grams per kilogram; h, hour; mg/dL, milligrams per deciliter.*

**TABLE 3:** Reversal learning summary from reviewed studies outlining behavior task, effect, methodology and subjects.

Behavior Task	Effect	Voluntary/Non-Voluntary	Method of Administration	Alcohol Exposure (days)	Abstinence before test	Intake (g/kg)	Species: Genotype	Sex: M/F	Age of exposure	References
Reversal- Barnes Maze	√	Non-voluntary	Gavage	5	11–13 days	5 g/kg	Rat: Wistar	M	Adult	Marszalek-Grabska et al., 2018
Reversal- Barnes Maze	×	Non-voluntary	Gavage	∼ 30	3 weeks	5 g/kg	Rat: Sprague Dawley	M	Adolescent	Fernandez and Savage, 2017
Reversal- Barnes Maze	√	Non-voluntary	IP + Gavage	1 or 5	11–13 days	5 g/kg	Rat: Wistar	N/S	Adult	Gibula-Tarlowska and Kotlinska, 2020
Reversal- Barnes Maze	√	Non-voluntary	Vapor	21–28	5–7 days	N/S	Mouse: C57BL/6J	M	Adult	Varodayan et al., 2018
Reversal- MWM	×	Non-voluntary	Gavage	4	4.5 days	8.8 g/kg (F) 8.3 g/kg (M)	Rat: Long-Evans	M + F	Adult	Maynard et al., 2018
Reversal- MWM	√	Non-voluntary	Gavage	30	25 + days	5 g/kg	Rat: Wistar	M	Adolescent	Vetreno et al., 2020
Reversal- MWM	√	Non-Voluntary	Liquid Diet	49	22–24 days	9 g/kg (Ad), 7 g/kg (Ag)	Rat: Sprague Dawley	M	Adult + Aged	Novier et al., 2016
Reversal – Olfactory	√	Non-voluntary	Gavage	25	24 h or 3 weeks	5 g/kg	Rat: Sprague Dawley	N/S	Adolescent, Adult, Aged	Fernandez et al., 2017
Reversal – Olfactory	IP: √ Gavage: ×	Non-voluntary	IP + Gavage	14–42	5–7 days	3–5 g/kg	Rat: Sprague Dawley	M	Adult	Badanich et al., 2016
Reversal – Operant	√	Non-voluntary	Gavage	4	5 days	11–15 g/kg/day	Rat: Wistar	M	Adult	Cippitelli et al., 2017
Reversal – Probabilistic	√	Non-voluntary	Gavage	32	32 days	5 g/kg	Rat: Sprague Dawley	M + F	Adolescent	Galaj et al., 2019
Reversal – Touchscreens	√	Voluntary	2BC	180	3 days	∼6 g/kg	Rat: Long-Evans	M	Adult	Charlton et al., 2019

*M, male; F, female; MWM, Morris water maze; Gavage, intragastric or oral gavage; IP, intraperitoneal injection; Vapor, vapor inhalation chamber; Liquid Diet, Lieber DeCarli liquid ethanol diet; Drinking Water, alcohol in drinking water; DID, drinking in the dark; 2BC, two-bottle choice; 4BC, four-bottle choice; Self-Admin, Oral self-administration; N/S, Not explicitly stated; g/kg, grams per kilogram; h, hour; mg/dL, milligrams per deciliter; Ag, aged; Ad, adult.*

**TABLE 4:** Set-shifting summary from included studies outlining behavior task, effect, methodology and subjects.

Behavior Task	Effect	Voluntary/Non-Voluntary	Method of Administration	Alcohol Exposure (days)	Abstinence before test	Intake (g/kg)	Species: Genotype	Sex: M/F	Age of exposure	References
Set-Shifting – Operant	√	Non-voluntary	Gavage	30	3 weeks	5 g/kg	Rat: Sprague Dawley	M	Adolescent	Fernandez and Savage, 2017
Set-Shifting – Operant	√	Non-voluntary	Gavage	22	20–25 days	4 g/kg	Rat: Sprague Dawley	M + F	Adolescent	Varlinskaya et al., 2020
Set-Shifting – Operant	√	Non-voluntary	Vapor	35	2–10 days	N/S	Rat: Long-Evans	M	Adult	Natividad et al., 2018
Set-Shifting – Texture and Odor	×	Non-voluntary	Vapor + Drinking Water	29	4–5 days	0.75–1 g/kg/h	Mouse: C57BL/6J	M	Adult	Rodberg et al., 2017
Set-Shifting – Texture and Odor	√	Voluntary	Self-Admin	30	none	0.5–0.8 g/kg/h	Rat: Wistar	M	Young adult (250g), N/S	Zhang et al., 2019
Set-Shifting- Plus-maze	√	Non-voluntary	Vapor + 2BC	37–54	2–3 days	2BC: 1.5–2.5 g/kg/2 h	Mouse: C57BL/6	M	Adult	Pradhan et al., 2018

*M, male; F, female; Gavage, intragastric or oral gavage; IP, intraperitoneal injection; Vapor, vapor inhalation chamber; Drinking Water, alcohol in drinking water; 2BC, two-bottle choice; Self-Admin, Oral self-administration; N/S, Not explicitly stated; g/kg, grams per kilogram; h, hour; mg/dL, milligrams per deciliter.*

The wide range of methodology used across studies provides a valuable insight into how these varying factors impact alcohol intake and resulting cognition. Unfortunately, the domains outlined above are not always consistently reported, making it difficult to compare studies accurately, and difficult to interpret discrepancies in results. Often studies neglect to report abstinence period or intoxication level, two fundamental factors when looking at alcohol use. It would be useful to create reporting guidelines to ensure results are consistent and comparable in the future. Limitations in journal length may be a factor impacting the limited reporting, but as reproducibility is fundamental in science and without it studies cannot be clearly interpreted or replicated, this is providing a barrier to translation (Perry and Lawrence, 2017).

## Methods of Administration

Across the literature there is substantial variability in methods used to administer alcohol. These can broadly be divided into non-voluntary/forced, or voluntary. Even within these two categories, there are numerous different modes through which alcohol can be administered. These differences arise from the need to encourage high rates of intake, however, it is important to consider the confounding effects they may have. No doubt due to factors such as control over intake levels and timing, and issues with palatability, non-voluntary methods constitute majority of research designs. One of the most common non-voluntary methods is intragastric gavage administration (e.g., Majchrowicz protocol), where animals have alcohol (usually 3–7 g/kg/day) administered by passing a feeding needle through the mouth and into the esophagus. The Majchrowicz protocol in particular models binge intake, and involves 12 gavage administrations over 4 days. Initial dose is 5 g/kg, and subsequent doses are determined based on physical indications of intoxication in the subject (Faingold, 2008). Of course, gavage administration is not restricted to this schedule, and can be administered at doses determined by the experimenter. This method is precise and mimics the way alcohol would be ingested by humans. However, it can induce esophageal damage (Jones et al., 2016), and is a stressful procedure for animals – particularly with the frequency required to model chronic alcohol use disorders. Another forced method is intraperitoneal (i.p.) injection of alcohol, where animals are administered alcohol *via* needle directly into the intraperitoneal cavity. This route has fast and complete absorption when done correctly, however relies on experimenter accuracy. It is also uncomfortable and stressful for the animals, particularly with the restraint handling (Huang et al., 2015). These two forced methods of administration, gavage and i.p. injection, have been shown to produce similar high BAC across timepoints, achieving peak BACs up to ∼350–400 mg/dL depending on the dose (Ghosh Dastidar et al., 2018). Lastly, there is a forced administration method that attempts to reduce handling stress, namely ethanol vapor chamber. Here the animals are exposed to alcohol *via* inhalation in an airtight container. Alcohol bypasses initial metabolism and rapidly reaches arterial circulation and the brain, potentially increasing the risk of addiction; although this has not been extensively studied (Maclean et al., 2017). The dose is gradually ascending, producing blood alcohol concentrations of around 175 ± 25 mg/dl (Rogers et al., 1979; Becker and Lopez, 2016; Peterson et al., 2017) and sometimes even >500 mg/dl, depending on age, sex and genotype (Glover et al., 2021). Pyrazole, an alcohol dehydrogenase inhibitor, is administered to slow the metabolism of alcohol in mice (Goldstein, 1975), although this is not necessary in rats (Ferko and Bobyock, 1977; Wang et al., 2012). This method reduces handling stress for animals, while tightly controlling dose and timing (particularly that of abstinence) and reducing the confound of calories.

Less forceful non-voluntary methods, such as the ethanol liquid diet (Lieber-DeCarli model-, Lieber et al., 1989), allow animals to have free access to a nutritional liquid that contains alcohol, but with no other food or liquid available, producing peak blood alcohol levels of around 100 to 160 mg/dl (Lieber et al., 1989; Guo et al., 2018). The Lieber-DeCarli model does come with the caveat that the diet is high in fat, which can lead to cognitive deficits independent of alcohol (Sketriene et al., 2021), and must be controlled for. Alternatively, drinking water alone might be replaced with an alcoholic solution, with *ad libitum* food, although this produces lower blood alcohol levels of around 70 mg/dl (Lieber et al., 1989; Brandon-Warner et al., 2012). Although in these cases intake is initiated by the subject, the lack of *ad libitum* water (and in some cases food) means that consumption is still obligatory.

The drinking in the dark paradigm relies on a similar principle, but consumption is more voluntary. Water is replaced with alcohol for only 2 h, starting 3 h after the dark cycle begins. This continues for 3 days, then on the 4th day, access is extended to 4 h (Rhodes et al., 2005). This limited access schedule produces binge-like consumption, particularly on the final day, producing blood alcohol levels up to 80–100 mg/dL in mice (Thiele and Navarro, 2014; Bauer et al., 2021) and between 20 and 50 mg/dl in rats (Holgate et al., 2017).

Lastly, there are completely voluntary consumptions models. These usually involve *ad libitum* access to food and water with the addition of an alcohol bottle (up to 20%), creating a two-bottle choice. In some cases, there are three alcohol bottles of varying concentrations in addition to the water bottle, creating a four-bottle choice. This alcohol access is usually for 24 h, dispersed intermittently across a week and producing BACs greater than 80 mg/dl in rats (Simms et al., 2008; Carnicella et al., 2014) and mice (Hwa et al., 2011); although lower BACs are sometimes reported in rats (40 mg/dl; Holgate et al., 2017). Sweeteners and other additives may also be used to increase palatability of alcohol and encourage intake. However, these may themselves have an effect on behavior and cognition if consumed over extended periods (Erbaş et al., 2018; Jacques et al., 2019). In particular, high-calorie additives such as carbohydrates have been shown to cause cognitive deficits (Hawkins et al., 2018; Sketriene et al., 2021). It is therefore important that if sweeteners are used, they are controlled for.

These various different methods of administration clearly have different strengths and weaknesses. Where alcohol intake is non-voluntary, the experimenter has control over the dose administered, and furthermore can achieve BACs that are consistently biologically relevant (Rogers et al., 1979; Faingold, 2008; Ghosh Dastidar et al., 2018). However, these methods may pose significant handling stress on the subject. Similarly, where alcohol is compelled due to a lack of alternate food or water sources, this would presumably precipitate a physiological stress response. Given the extensive literature indicating that stress can impact cognitive decline (e.g., see Hupalo et al., 2019a), and the fact that stress interacts with the effects of alcohol (Koob, 2015; Tunstall et al., 2017), this may in fact confound any findings produced. Voluntary intake is less stressful but affords the experimenter less control and leads to lower levels of intake (Simms et al., 2008; Thiele and Navarro, 2014). Over the following sections, we will review recent literature looking at cognitive decline following chronic alcohol exposure using these methods. We will split each section into non-voluntary and voluntary methods, and further examine the effects of other factors such as length of exposure and abstinence period. Unless stated otherwise, all studies were undertaken in males. Many of the studies reviewed employed other interventions, or probed mechanisms. Where this was the case we looked only at the control group, as we were only interested in the effect of alcohol on cognition for the purpose of this review. We hope to be able to provide at least some insight into the advantages and disadvantages provided by different research methods for understanding the effects of chronic alcohol, and that these insights might in the future be used to design improved studies for investigating the mechanism of alcohol-induced cognitive decline, as well as potential interventions.

### Spatial Learning and Retrieval

Spatial representation and navigational skills are commonly reported domains affected by alcohol in both humans and rodent preclinical studies (Silvers et al., 2003). Corollary to this, the hippocampus, known to be a key neural locus for processing spatial information and memory (Silvers et al., 2003; Buckley, 2005), is particularly vulnerable to alcohol insult (Staples and Mandyam, 2016). It is worth noting that the hippocampus is also sensitive to the effects of chronic stress (Kim et al., 2015), meaning that this confound might be particularly salient when considering how schedules of alcohol access mediate alcohol-induced effects on cognitive domains that involve spatial navigation.

Findings from manuscripts that assess spatial learning and memory can be seen in Table 1. These are often assessed using the Barnes maze (Barnes, 1979) or the Morris water maze (Morris, 1984), both of which require the animal to remember the location of a target zone with the assistance of distal visual cues around the testing area. Barnes maze typically involves a round platform under bright house lights, with holes around the perimeter, one of which will lead to an escape box. There is often an aversive aural stimulus playing while the subject is in the arena, which terminates upon entry into the escape box (Gibula-Tarlowska and Kotlinska, 2020). In the Morris water maze, animals are placed in a large circular pool of water and required to escape onto a hidden platform (Harrison et al., 2009). Both tests rely on the animals’ desire to escape an aversive stimulus or environment and are therefore inherently stressful.

The radial arm maze has a similar premise to the Barnes maze and Morris water maze but relies on positive reinforcement. This has up to 8 arms radiating from a central platform, each terminating with a food cache that is not visible from the center (Brown et al., 2007). The subject must learn which arms contain the filled cache. Working memory is inferred through their ability to avoid visiting an arm where food has already been consumed (where all arms are baited), while reference memory can be assessed through baiting only a single arm and measuring the length of time the subject takes to learn this location.

Alternatively, mazes such as T-maze and Y-maze capitalize on rodents’ natural inclination to explore novel arms (reference and working memory). For the T-maze, the animals are placed in the “bottom” of a T-shaped maze with one arm blocked off. The animals are later returned to the maze with no arms blocked off, and the time spent in the novel arm is measured. The Y-maze uses the same protocol, but with the gentler angles of a Y-shape. Spatial memory can also be measured using the object-in-place task, which requires subjects to investigate 2 objects based on spatial cues. While the mouse is not in the area, one of the objects is moved. Again, the rodent’s inherent preference for novelty means that they will spend more time investigating the novel location object if they recall the objects’ original locations (Denninger et al., 2018).

Binge-like intoxication, where high doses of alcohol are administered *via* intragastric gavage, is associated with deficits in the Barnes maze and Morris water maze, although the findings are mixed. 5 days of repeated gavage at 5 g/kg produced impairments in retrieval of the location of the Barnes maze escape box when rats were tested after 11 days of abstinence (Gibula-Tarlowska and Kotlinska, 2020). On the other hand, a similar regime (5 days gavage at 5 g/kg/day administered to rats post-training and followed by 11 days abstinence) had no effect on primary escape latency or the number of errors in the probe trial (Marszalek-Grabska et al., 2018). Both studies do, however, find impairments in the reversal phase of this task, which is discussed in the following section.

A more chronic regime of intragastric gavage across the adolescent period in male rats (16 gavages with a schedule of 2 days on, 2 days off, at 5 g/kg dose), failed to show impairments in spatial memory in the Barnes maze (Fernandez and Savage, 2017). In this study, however, unlike in the previous two mentioned, training occurred after completion of the alcohol regime and 21 days of abstinence (longer than previous studies), reflecting intact capacity to acquire rather than retrieve the memory for location of the escape box. This distinction cannot be the only relevant manipulation, however, because adolescent rats administered 5 days of gavage at a lower dose (1.5 g/kg/daily) followed by 15 days abstinence showed spatial reference memory deficits during acquisition of the radial arm maze task (Loxton and Canales, 2017).

Mice exposed to vapor chamber alcohol for 3–4 weeks followed by 5–7 days abstinence (BAC around 208.8 ± 14.3 mg/dL) did not show any differences in initial acquisition of Barnes maze, but interestingly, were faster with reacquisition than controls (Varodayan et al., 2018). This could reflect increased motivation; and it’s worth nothing in this regard that the abstinence period was shorter in this study than in the gavage binges described above.

The Morris water maze also provided mixed findings regarding the acquisition of spatial representation. For example, adolescent exposure, where male rats were gavaged daily (5 g/kg/day) for 30 days, followed by 25 days abstinence (to adulthood), did not lead to deficits in task acquisition (Vetreno et al., 2020). However, a similar exposure period of 28 days but with a lower alcohol dose (3 g/kg) was sufficient to impair spatial memory in rats after 24 h abstinence (Pamplona-Santos et al., 2019). It is probable that the length of abstinence before testing may underlie these differing results. In other words, alcohol-induced deficits in acquisition of a Morris water maze task may be transient.

A single four-day Majchorwicz binge gavage regime in adult female (≈8.8 g/kg/dose) and male (≈8.3 g/kg/dose) rats, followed by 4.5 days abstinence, produced deficits in acquisition of spatial learning in females, but not in males (Maynard et al., 2018). This highlights possible female vulnerability. Furthermore, the same regime but at a higher dose (11–15 g/kg/dose), followed by a similar abstinence period of 5 days, was sufficient to produce spatial learning deficits in male rats (Cippitelli et al., 2017) suggesting that alcohol-induced deficits are dose-sensitive. Consistent with this, 1, 5, and 10 days gavage exposure at a lower dose of 5 g/kg once daily, followed by 5 days abstinence, showed no observed effect of alcohol on spatial learning or memory in mice (Wang et al., 2018). Similarly, 5 days exposure (4 g/kg, administered i.p.) and 16 days abstinence in neonate rats produced no effect of alcohol on spatial learning/memory (Swart et al., 2017).

Longer exposure periods of gavage were more likely to produce deficits. Fourteen days with varying concentrations of 12.5, 25, and 50% alcohol, with no abstinence period between exposure and test, led to impaired spatial learning at the two higher doses in both female and male mice (Xing and Zou, 2018). However, the lack of abstinence could mean the acute effects of alcohol may present a confound in this study. A lower dose (4 g/kg/day) did produce deficits in rats where exposure period was longer: gavage at this dose for 30 days, and then i.p. injection (1 ml/kg of 20% alcohol) for 30 days delayed acquisition of Morris water maze, and decreased the time spent in target quadrant at test (Vaghef et al., 2019). Similarly, male rats given 56 days exposure (10 g/kg/day *via* gavage) and 11 h abstinence, displayed impaired spatial learning and memory (Chen and Hu, 2017), as did male mice exposed for 42 days (5 mg/kg, i.p.; with length of abstinence undefined) (Ikram et al., 2019). In summary, forced alcohol intake *via* both intragastric gavage or i.p. injection has been reported to impact spatial memory, but this is a dose-dependent and possibly transient effect in both cases.

Lieber DeCarli liquid diet involves providing subjects with a liquid diet in place of lab chow, and adding alcohol to it for the experimental group. Spiking food source with alcohol might be considered less stressful than gavage or i.p. injection, however here alcohol consumption is still coerced, since no alternative is available. Lieber DeCarli liquid diet over 28 days followed by no abstinence was sufficient to impair acquisition and retrieval of the Morris water maze task in mice (Wang et al., 2017). On the other hand, no deficits in acquisition were found in a study where liquid alcohol diet was provided for 7 × 7 day cycles, with 24 h of withdrawal between each cycle. This schedule led to consumption of on average 9 g/kg/day in adults and 7 g/kg/day in aged adults. Rats were then left in abstinence for 21 days before being given a relatively low challenge dose of 1.5 g/kg, administered *via* i.p. No deficits were found in either age group (Novier et al., 2016).

Where consumption was on a purely voluntary base, it seems that length of exposure was again important. 48 days of access *via* a four-bottle choice (intake ≈20 g/kg/day) did lead to impaired spatial memory in male mice (Liu et al., 2019). Here, there was no protracted abstinence, although sufficient time elapsed for the acute effects of withdrawal to abate. Similarly, two-bottle choice for 43 days in mice (intake was 15 g/kg) (Rajput et al., 2017) or 84 days in rats (Dwivedi et al., 2018; Pandey et al., 2020) also led to impaired spatial memory. However, a shorter exposure period of 28 days (7–11 g/kg) in mice, followed by no abstinence, did not lead to deficits in spatial memory (Pant et al., 2017). This further supports that longer exposure and higher doses lead to worse cognitive impairments.

T-Maze and Y-maze are similar tasks that measure an animal’s ability to navigate through a familiar three-armed apparatus. Here, a 4-day binge exposure *via* the Majchorwicz gavage method (adjusted dose given every 8 h) produced deficits in spatial working memory in both male and female rats (West et al., 2018). Rats were abstinent for 4 days before testing. On the other hand, mice that had alcohol exposure *via* a combination of non-voluntary administration (vapor chamber) and voluntary administration (two-bottle choice) for 37–54 days did not show impaired spatial working memory (Pradhan et al., 2018). In this study, mice had access to alcohol for 2 h a day and drank at a low rate of 1.5–2.5 g/kg across this period. There was a short abstinence period – 2–3 days – prior to testing. However, a longer exposure of 6 months in aged mice, where drinking water was replaced with increasing concentrations of alcohol (i.e., forced drinking) did produce deficits to spatial working memory, although interestingly these only emerged following 1 or 6 weeks abstinence, and were not present if mice were tested immediately following termination of alcohol access period (Dominguez et al., 2016).

Alcohol exposure consistently produced deficits in Y-maze performance. A 28-day forced alcohol exposure *via* spiked drinking water impaired spatial and learning memory in rats (Du et al., 2019). In mice, a longer exposure of 42 days (5 mg/kg daily i.p.) (Ikram et al., 2019), or 84 days (George et al., 2018), also impaired spatial working short-term memory. In the latter study it is unclear how alcohol was administered, however the dose applied (1.5 g/kg) was low. In all three studies, abstinence length following exposure was not stated, so it is unknown whether animals were acutely intoxicated or in withdrawal, which could affect interpretation of results.

The final task that measures spatial ability is a variation of novel object recognition. In its basic form, novel object recognition probes an animal’s ability to distinguish between familiar and unfamiliar stimuli, and is not primarily a spatial task. However, it can be adapted to probe spatial recognition. For example, in the object-in-place task, one (or two) of a number of familiar objects is moved, and spatial memory is operationalized as the time spent inspecting the object in the new location as opposed to the object in a familiar location. Performance in this task was impaired in female rats following 15 days of forced alcohol consumption *via* the Lieber-DeCarli liquid diet (increasing concentrations of alcohol up to 15%) (King et al., 2020). Here, the abstinence period was only 7 h, which would put the mice in acute withdrawal, magnifying the effect on cognition. However, voluntary access with a longer period of abstinence also produced deficits. Mice that received 28 days access to alcohol for 4 cycles of drinking in the dark, consumed up to 4–6 g/kg over these sessions. When tested after 2 weeks of abstinence, alcohol-exposed mice did not distinguish between the moved and unmoved object (Rico-Barrio et al., 2019).

In summary, chronic alcohol usually produces deficits in spatial learning and memory following chronic alcohol exposure in both voluntary and non-voluntary preparations. Where deficits were found in voluntary models, levels of intake were unusually high (20 g/kg/day, Liu et al., 2019 and 15 g/kg/day, Rajput et al., 2017). In fact, across the board, where deficits are not found, it seems due to a lower alcohol dose. Sweetening alcohol with saccharine did appear to encourage intake (e.g., Dwivedi et al., 2018; Pandey et al., 2020), which in turn meant that deficits were more likely to be found.

Abstinence may also be a factor, since for both Morris water maze and Barnes maze, deficits were less likely to be found in studies where there was period of abstinence between exposure and test. This suggests the deficit is transient (Novier et al., 2016; Swart et al., 2017; Wang et al., 2017, 2018). This was not the case for studies that tested spatial working memory using T-maze and Y-maze protocols (Dominguez et al., 2016; Pradhan et al., 2018). Furthermore, other inconsistencies between the studies make it difficult to infer the effect of any one manipulation. Nevertheless, the role of abstinence in uncovering or recovering cognitive effects of chronic alcohol is an interesting question that may be worth systematic investigation in the future.

### Working Memory and Discrimination

Discrimination tasks measure an animal’s ability to respond differently to different stimuli. They are thought to rely on declarative memory, recognition memory and habit memory, which are mediated largely by the dorsal striatum (Broadbent et al., 2007). We have summarized results from studies looking at discrimination-based tasks in Table 2. Discrimination tasks can be based on object, odor, auditory stimuli, pattern, or novelty. They can be measured in operant tasks, where the subject needs to select a particular object from a choice of (usually) two discrete objects to obtain a reward (e.g., Charlton et al., 2019). They can also be measured by the relative amount of time spent investigating a novel object versus one that is familiar, which reflects not only the subject’s ability to discriminate between two objects, but also their ability to remember objects that they have encountered in recent experience (e.g., Leger et al., 2013; Golub et al., 2015).

The overwhelming majority of studies over the last 5 years that have examined the effect of chronic alcohol on discrimination learning and retrieval have done so using the novel object recognition task. This test is widely used to assess the effects of both voluntary and non-voluntary models of alcohol administration. As with the previous behavioral tests reviewed, the findings are mixed.

Seven days of administration *via* gavage (3 g/kg/day) followed by 24 h abstinence was sufficient to produce a novel object recognition deficit in rats (Geiss et al., 2019). Likewise, mice exposed to a schedule of 16 repeated i.p. injections across adolescence (2 per day at 1.25 g/kg, 2 days on, 2 days off) showed an impaired discrimination index, where testing occurred in adulthood, approximately 40 days later (Ledesma et al., 2017). On the other hand, a longer (28 day) exposure across adolescence (3 g/kg/day) followed by only 24 h abstinence did not lead to an impairment in novel object recognition compared to controls, although performance was impaired compared to rats that were forced to exercise on a treadmill alongside alcohol treatment (Pamplona-Santos et al., 2019). In another experiment looking at adolescent exposure in rats, 16 gavages (5 g/kg/dose; with 2 days on, 2 days off), followed by 21 days abstinence, also failed to produce novel object recognition deficits (Fernandez and Savage, 2017). With neonatal alcohol exposure, 5 days of i.p. injections (4 g/kg/day) followed by 16 days abstinence had no effect on rats (Swart et al., 2017).

It is unlikely, however, that these differences are due to age specific effects. 30 days adolescent exposure (5 g/kg/day), followed by a longer abstinence of 90 days, did reduce discrimination ratio relative to controls in both male and female rats (Vetreno et al., 2016). Likewise, four cycles of binge exposure across adolescence (3 g/kg i.p. daily for 4 days, followed by 3 days abstinence) produced deficits in discrimination ratio in alcohol-exposed rats when tested in adulthood, 8 days later (Sanchez-Marin et al., 2017). This shows that adolescent resilience does not underlie differences in results, and indeed even a one day of exposure (2 × 3 g/kg, administered i.p.) followed by 48 h abstinence, in late adolescent rats, led to impaired novel object recognition (Drissi et al., 2020). Thus, where alcohol is administered *via* gavage or i.p. injection, the impact of dose, age of exposure and length of abstinence on the ability to recognize and discriminate between a familiar and a non-familiar object is difficult to decipher.

Vapor chamber exposure consistently led to novel object recognition deficits. This was evident in mice exposed for 28 days (abstinence not stated) (Nie et al., 2018), as well as in mice exposed for 37–54 days, with a combination of voluntary intake and vapor chamber exposure (Pradhan et al., 2018). In addition, mice that had 84 days alcohol exposure, where the administration method was not stated, also showed impaired discrimination in this task (George et al., 2018). Finally, it’s worth noting that performance in this task is context-dependent, and this allows researchers to test an animal’s ability to distinguish between contexts following alcohol exposure. Animals are exposed to two different contexts, each housing a unique pair of identical objects. After a delay, animals are tested by re-exposure to the context, but holding a familiar and novel object; and interaction with the novel object for that context is measured. Mice with 29 days alcohol exposure (0.75–1 g/kg/h) *via* vapor chamber and drinking water, followed by 72 h abstinence, demonstrated reduced discrimination of object/context pairings (Rodberg et al., 2017), demonstrating that contextual discrimination is also impacted by alcohol.

In voluntary models, mice showed deficits following 43 days of two-bottle choice across adulthood (15 g/kg/session; no abstinence, Rajput et al., 2017), and following 28 days drinking in the dark across adolescence (4–6 g/kg/2 h session; 2 weeks abstinence, Rico-Barrio et al., 2019). However, 28 days exposure (7–11 g/kg/session; no abstinence) did not have an effect on mice (Pant et al., 2017). On the other hand, when this was studied in adolescent rats allowed access to four-bottle choice, 49–56 days of alcohol access *increased* the discrimination index when tested within the 1st week of abstinence. This meant that animals spent more time with the novel object than the familiar object (Silva-Peña et al., 2019) and suggests improved discrimination and working memory following alcohol exposure. Notably, the drinking rate was substantially lower in this sample, with rats consuming on average 2.2 g/kg/24 h. While this is the only discrimination-based study that found improved performance in the alcohol-exposed group, it is consistent with reports that low/moderate alcohol might improve cognitive performance in certain tasks (Xu et al., 2017; Rehm et al., 2019).

There are other ways to study discrimination learning. For example, the passive avoidance task is a fear-motivated test used to evaluate learning and memory, and probes an animal’s ability to distinguish between two contexts. Mice with 84 days alcohol exposure (method not stated, abstinence not stated) showed long-term memory dysfunction compared to controls in the passive avoidance task. These rats spent a relatively greater amount of time in the treat-associated chamber, and showed longer latency to leave that side (George et al., 2018).

Subjects can also be trained to discriminate between two stimuli (olfactory, visual), where a response to one of the two is rewarded. In fact, most studies that examine discrimination in this way do not find an effect for chronic alcohol. For example, in adult rats, neither i.p. administration of 4 g/kg/day over 6 weeks, nor intragastric gavage at the same dose, produced deficits in acquisition of an olfactory discrimination task (Badanich et al., 2016). In each case, there was a 1-week abstinence period prior to test. This study also examined the effect of higher doses using intragastric gavage, however, no dose was sufficient to incur a discrimination deficit. A shorter but higher regime, where 5 g/kg/dose alcohol given in 13 gavage sessions over 25 days, did produce a deficit in olfactory discrimination in adult rats, who required more trials to reach criterion (Fernandez et al., 2017). However, there was still no effect evident where alcohol was given across early or late adolescence. Similarly, a 4-day Majchorowicz binge in rats did not influence acquisition in an operant discrimination task (Cippitelli et al., 2017), nor did six-month voluntary access *via* two-bottle choice, where rats reached consumption rates of 6 g/kg/24 h (Charlton et al., 2019).

### Reversal Learning and Set-Shifting

Impaired behavioral flexibility is in fact one of the hallmarks of substance use disorder and is often attributed to impaired function in prefrontal neural regions (Izquierdo et al., 2017). Behavioral flexibility reflects the ability to adjust behavior in response to the conditions of the current situation (Charlton et al., 2019), and can be assessed in animal models by probing their ability to adapt to a reversed condition in a task already acquired, or to shift their strategy for finding a reward after a rule change in an operant task.

#### Reversal Learning

Most of the tasks described above can include a reversal phase, where the contingencies of reward or punishment are changed, or the spatial configuration of the testing arena is altered so that the subject must change their response accordingly. Manuscripts reporting a reversal deficit are summarized in Table 3. Often, even if no deficit is reported for the acquisition phase, chronic alcohol produces deficits during reversal; although this is more common for discrimination tasks than for spatial tasks. For example, in the Barnes maze, reversal learning involves altering the location of the escape hole, usually to the opposite side of the field. Deficits can emerge at this stage of the task, although gavage administration has mixed effects. 5 gavage exposures (5 g/kg each) followed by 11–13 days abstinence did not affect rats’ acquisition, but did result in reversal learning deficits (Marszalek-Grabska et al., 2018). Following similar alcohol administration (5 gavages, 5 g/kg, followed by 11–13 days abstinence), reversal learning deficits were observed in rats. However here there were also impairments with the initial acquisition so the reversal deficits may reflect overtraining in the original learning to help all subjects reach criterion (Gibula-Tarlowska and Kotlinska, 2020). In contrast, 16 gavage exposures (5 g/kg) in adolescent rats followed by 21 days abstinence did not induce reversal learning deficits (Fernandez and Savage, 2017). Interestingly, vapor chamber administration led to improved reversal learning and set-shifting in mice with 3–4 weeks exposure (BACs around 208 mg/dL) followed by 5–7 days abstinence (Varodayan et al., 2018), which could be explained by increased motivation, as these same animals also had faster reacquisition than controls.

In the Morris water maze task, reversal learning results are similarly mixed. Here again, the reversal phase involves altering the configuration of the pool so that the concealed platform is in the opposite quadrant. 4 days Majchrowicz binge followed by 4.5 days abstinence did not cause any reversal learning deficits in male or female rats (Maynard et al., 2018). However, a longer gavage exposure led to reversal learning deficits in rats after 30 days adolescent exposure (5 g/kg/day, two days on, two days off) followed by 25 days abstinence and testing in adulthood (Vetreno et al., 2020). These differences could be due to the different ages of exposure, however, as previously, the most parsimonious explanation is that longer or larger exposure led to greater likelihood of impairment. Supporting this, Lieber DeCarli liquid diet impaired reversal learning following alcohol exposure in adult rats that consumed 9 g/kg/day and aged adults (7 g/kg/day) after 49 days exposure with weekly 24-h withdrawals. Note that here they were tested after 21 days abstinence followed by a single alcohol challenge of 1.5 g/kg which was administered i.p. 24 h before behavioral testing (Novier et al., 2016), which suggests that the difference is more likely due to varied exposure length.

In an olfactory discrimination task, 13 gavage administrations over 25 days impaired reversal learning regardless of age at exposure (early adolescent/mid adolescent/adult) (Fernandez et al., 2017). These rats were tested in adulthood, following up to 3 weeks of abstinence, and the effect was most evident upon first reversal. Furthermore, blood alcohol concentration reported correlated with the number of trials required to reach reversal criterion. In this task, there is some systematic evidence that method of administration is important. When administered i.p., but not by gavage, 4 g/kg/dose produced reversal deficits in rats in an odor discrimination task (Badanich et al., 2016). Increasing number of gavage administrations to 2 per day, escalating the dose administered to 5 g/kg, and decreasing abstinence from 7 to 5 days all failed to produce an effect (Badanich et al., 2016). This is the only study to our knowledge that systematically tests the effects of different administration, although notably the range of dose and timing is narrow.

Deficits in operant reversal learning task were found in rats following 4 days gavage (11–15 g/kg) and 5 days abstinence (Cippitelli et al., 2017). Gavaged alcohol also produced deficits in reversal in a more complex operant probabilistic task (Galaj et al., 2019). Here, male and female rats received 16 gavage doses at 5 g/kg over 30 days across adolescence. Both “correct” and “incorrect” levers were reinforced, however the correct lever was reinforced at a higher ratio. The designation of correct vs incorrect furthermore reversed within session once a particular criterion was attained. Both males and females showed deficits in this task, shown by greater response latencies. The deficits were more pronounced for male rats, who required a greater number of trials to achieve three reversals (Galaj et al., 2019). Reversal of a simpler pairwise discrimination task using a touchscreen platform was impaired in rats that had consumed alcohol voluntarily under two-bottle choice conditions for 6 months (6 g/kg/day, 3 days/week) (Charlton et al., 2019).

### Set-Shifting

Set-shifting requires animals to inhibit an earlier learned response in order to learn a new one, similar to reversal learning. However, set-shifting is often more complex than reversal. For example, in an operant task, rules such as “respond to the lever under the illuminated light” or “always respond to the right-hand lever” might be established. Throughout the task, these rules are changed, and the subject must learn the new conditions to obtain the reward. Like reversal learning, this is a measure of behavioral flexibility and is sensitive to the effects of chronic alcohol. We have summarized findings from studies reporting on a set-shifting task in Table 4.

In non-voluntary models, 16 gavages (2 days on and 2 days off – 5 g/kg/dose) during adolescence, followed by 21 days abstinence, led to an increased number of trials required to set-shift in rats (Fernandez and Savage, 2017). 22 days of gavage (4 g/kg/dose) at early and late adolescence, in male and female rats, followed by 20–25 days abstinence, also produced a set-shifting deficit, though only increased regressive errors were found – that is performing an error despite having previously performed a correct response. Further, this was only evident in males that were exposed in early (not late) adolescence (Varlinskaya et al., 2020). Vapor chamber exposure over 35 days followed by 2–10 days abstinence produced a deficit in rats that was manifested as greater number trials required to reach criterion as well as greater number of errors made after the shift (Natividad et al., 2018).

Plus-maze, named for its shape, can also be used for set-shifting. Here the subject needs to find the baited arm using a set of rules, where there is shifting between a direction-based to a cue-based rule. There is only one study that looked at Plus-maze set shifting, which used a combination of vapor chamber alcohol exposure and two-bottle choice access to induce dependence in mice. Here, 37–54 days exposure produced deficits so that alcohol-exposed mice took longer to reach criterion under each rule set, and performed a greater number of errors than controls (Pradhan et al., 2018). A similar mixed schedule over approximately 4 weeks did not produce set-shifting deficits in mice where the rule shifts were in a two-dimensional odor and texture discrimination task (Rodberg et al., 2017). Differences here may be due to slightly shorter exposure time and the lower intake levels in the volitional drinking phases. That said, rats that were trained to self-administer alcohol over 28 days, achieving intake rates of 0.5–0.8 g/kg/h, showed deficits in odor and texture-based set-shifting, with increased number of trials required to complete the task and a higher error rate (Zhang et al., 2019). Although intake levels were smaller, an important difference here is that the subjects (in this case rats) had to learn to perform a specific response to obtain alcohol. This type of schedule can lead to development of habits following changes to the dorsal striatum (Dezfouli and Balleine, 2012), which in turn may manifest as inflexible behavior. Another difference is that this study did not have an abstinence period, and as such, it is difficult to exclude the possibility that the acute effects of alcohol withdrawal may be a confound.

Together the evidence presented in this section strongly implies that behavioral flexibility (reversal or set-shifting) is reliably affected by chronic exposure to alcohol. This effect appears to be independent of whether administration is voluntary or non-voluntary. There are certainly sex-dependent effects (Galaj et al., 2019; Varlinskaya et al., 2020) that would be worthwhile to follow up. Furthermore, the effect seems more robust in adolescent populations (Fernandez and Savage, 2017; Varlinskaya et al., 2020), meaning that it may be worth investigating how regions that are not fully developed at adolescence, such as the prefrontal cortex (Kim et al., 2017), might be recruited during these tasks, and whether immaturity of brain circuits might underlie the differences observed.

#### Learning Tasks in Non-Human Primates

Non-human primates can also be trained in cognitive behavioral tasks to assess cognitive domains such as behavioral flexibility, learning and memory (Shnitko et al., 2017). In one such study (Shnitko et al., 2019), late adolescent/young adult male rhesus macaques (4–5 years of age) were trained on operant set-shifting tasks conducted using touchscreens and dependent on visual discrimination, changing the discrimination dimension between shape and color. Performance in this cognitive task was scored based on the ratio of errors per trials, session duration, and maximum set reached, and these comprised a session performance index. Following assessment on the set-shifting task, the rhesus monkeys were taught to self-administer alcohol, and once this was acquired, they were given free access to water and alcohol for 22 h per day, 7 days per week, for a total of 22 weeks. Based on intake, animals were divided into low or heavy drinkers. Heavy drinkers averaged 3.29 ± 0.2 g/kg/day (BAC of 102 ± 10 mg/dl), low drinkers averaged 2.03 ± 0.2 g/kg/day (BACs of 29 ± 4 mg/dl). Heavy drinkers had a significantly lower performance index than low drinkers, indicating reduced cognitive flexibility may be a predictive trait of a heavy alcohol drinker. The data also highlight the impact of individual differences in drinking patterns (Xu et al., 2017).

Another study, also in rhesus macaques (Shnitko et al., 2020) looked at retention of a cognitive task following alcohol exposure. Here, male rhesus monkeys aged 3.5–4 years (indicating adolescence), consumed escalating doses of alcohol (0.5, 1, and 1.5 g/kg) *via* limited access self-administration for 90 days (30 days per dose), or a calorie-matched control. They were trained on the same set-shifting visual discrimination task as above, and undertook the task before alcohol exposure, and following 90 days exposure and 18 h abstinence. To make the task more difficult, the size of the stimuli was reduced once baseline was re-acquired. No differences were observed between ethanol-exposed or control groups in baseline acquisition (pre-alcohol) or retention (post-alcohol). However, when the size of the visual stimuli was reduced, the alcohol-exposed animals performed increased errors compared to controls. This demonstrates reduced cognitive flexibility when faced with a novel change.

Performance in the novel object recognition task was also associated with alcohol consumption in adult male rhesus macaques (4–6 years old) (Chandler et al., 2017). Monkeys were trained in an operant self-administration protocol the same as above, then allowed an additional month of free access to water and alcohol for 3 h per day. Although novel object recognition did not predict alcohol intake (unlike set-shifting, above), it was impaired following high total alcohol intake. Specifically, alcohol history produced increased perseverative behavior but not an inability to detect or react to novelty. These deficits persisted a year into abstinence, demonstrating that chronic alcohol can have lasting impacts on cognitive function. Together, these findings show that the cognitive decline observed following alcohol exposure may be a combination of both pre-existing cognitive deficits (such as cognitive flexibility) and those incurred by alcohol use (such as novel object recognition).

#### Non-cognitive Behavioral Effects of Alcohol – Motor and Affect

Motor function can be altered following alcohol consumption. Although it is less common for this to persist beyond acute intoxication, chronic motor changes have been observed in rats (da Silva et al., 2018), and humans (Woods et al., 2016). While an in-depth review of motor and affect-related behavioral changes were beyond the scope of this review, the overall findings of included rodent studies have been briefly summarized here. As most behavior tests have a locomotor component (e.g., swimming speed in the Morris water maze), it is essential to assess motor function to ensure it is intact and not confounding results. Most studies carefully control for this. For instance, in the Morris water maze, there is often an introductory task requiring animals to swim to a visible platform to assess swim speed (Vaghef et al., 2019). When a behavior task does not have a specific motor function task, an additional one can be used, e.g., rotarod or beam walk (Nie et al., 2018; Rico-Barrio et al., 2019). These results have not been reviewed, however in all cases where motor function was assessed, it was found to be unimpaired compared to controls; hence confirming that the deficits observed are indeed cognitive. This is something worth continued consideration, as impaired motor function could be falsely interpreted as cognitive dysfunction.

Disrupted affect, such as anxiety and depressive symptoms, commonly occur with alcohol intake, with mixed findings regarding whether they precede or arise from alcohol use (McHugh, 2019). Withdrawal from alcohol is thought to increase anxiety- (Gajbhiye et al., 2018) and depression-like behavior (Pang et al., 2013) both of which can persist into abstinence (Gong et al., 2017). While many studies in this review did not specifically assess affect, those that did focused on anxiety and found mixed results – with anxiety-like behavior sometimes increased (e.g., Vetreno et al., 2016; Varlinskaya et al., 2020), and other times unchanged (e.g., Novier et al., 2016; Swart et al., 2017; Maynard et al., 2018; Rico-Barrio et al., 2019). Depressive symptoms, addressed in only two studies, were unchanged (Ledesma et al., 2017; Rico-Barrio et al., 2019). This is something that should be considered in the future since increased anxiety and/or depressive symptoms may affect performance in behavioral tasks (Moritz et al., 2018). It is worth noting that while results were mixed in forced models, voluntary models did not produce any changes to anxiety (Rico-Barrio et al., 2019), suggesting in addition to occurring in response to alcohol exposure, anxiety-like behavior may also be affected by the alcohol administration method.

## Discussion

Across the literature reviewed there was substantial variability both in methodology and in results observed, as well as in the clarity and detail of reporting. As a result, it is difficult to draw any definitive conclusions regarding the impact of voluntary versus non-voluntary intake on likelihood of deficits being incurred. Cognitive impairment was observed following both voluntary and non-voluntary chronic alcohol administration. It is worth noting, however, that the vast majority of studies did employ a non-voluntary form of administration, which tends to suggest that impairment is more reliably observed using this practice. Nevertheless, there are some other common factors that seem to impact the severity of the alcohol-induced deficits. These include dose, length of access and abstinence, age of exposure and sex of animals. These factors are worth considering when designing experiments that investigate potential interventions that might reverse or prevent alcohol-induced cognitive decline.

The amount of alcohol consumed, usually reported in grams per kilogram, and occasionally in blood alcohol concentration, seems to be a major factor impacting whether deficits are apparent. It is worth noting that grams per kilogram can be an unreliable metric, especially with administration methods relying on bottled alcohol, as the measurement is taken as the volume missing, not that ingested by the animals. This can be problematic as bottles can leak, leading to overestimation. With this caveat in mind, dose-dependent responses were evident, with higher intake leading to more consistent deficits. In some cases, the same method was insufficient to induce deficits at one dose, such as 8.4 g/kg/day (Maynard et al., 2018), and sufficient at a higher one of 15 g/kg/day (Cippitelli et al., 2017). This suggests there is a threshold required to induce deficits, however, the minimum dose required does seem to vary greatly. That said, where assessed using a different task, the same underlying cognitive ability might show different sensitivity to alcohol. For example, in the Morris water maze, a high dose of alcohol did not produce deficits in spatial learning (Maynard et al., 2018), whereas using an appetitive radial arm task, a much lower dose (1.5 g/kg/day) was effective (Loxton and Canales, 2017). These differences could be accounted for by varying ages of exposure, and length of abstinence, but may also be due to sensitivity of the behavioral task.

In addition to the amount of alcohol consumed, the length of access also appears to be a factor impacting severity of deficits. Chronic models, unsurprisingly, were more consistent in inducing changes than those that were shorter. Interestingly, however, in some cases a lower dose over a longer period was more likely to induce deficits than a higher dose over a shorter period. While 4 days of Majchrowicz binge (≈8.4 g/kg/dose) was insufficient to produce deficits in adult males (Maynard et al., 2018), 3 g/kg/day over 28 days was sufficient in adolescents (Pamplona-Santos et al., 2019). This difference could also be attributed to different ages of exposure. It could also be due to varying abstinence periods, since the deficit was seen when tested after 24 h of abstinence (Pamplona-Santos et al., 2019), but not after 4.5 days (Maynard et al., 2018). It seems a combination of sufficiently high dose, and sufficient duration, are required to produce lasting alcohol-induced deficits. However, there are still ongoing questions as to why cognitive deficits are seen in some cases and not in others, despite similar doses and administration schedules being used.

The length of time between alcohol access period and behavioral testing also appears to have an impact on outcome, although the relationship is varied. In many cases, 24 h or less of abstinence resulted in impairments that were not seen following longer abstinence periods, suggesting deficits are frequently transient and recovery may be possible. This is most clearly illustrated where two gavage schedules of similar length and dose produced a deficit in spatial memory in a Morris water maze task following 24 h (Pamplona-Santos et al., 2019) but not 25 days of abstinence (Vetreno et al., 2020). Potential withdrawal-induced stress may contribute to cognitive deficits (Koob, 2008) which then recover once acute withdrawal symptoms have passed. Unfortunately, however, deficits were not always recoverable with longer abstinence. For example, a longer exposure of 6 months in aged mice only saw spatial working memory deficits in the T-maze following 1- or 6-weeks abstinence but not when tested immediately after cessation of drinking (Dominguez et al., 2016). In addition, Vetreno and Crews (2015) found deficits in object recognition that persisted up to 108 days abstinence. The persistence of these deficits into abstinence could be due to the longer exposure period, leading to increased alcohol-induced damage. Since many studies did not state their withdrawal/abstinence period, it is challenging to know precisely what role it is playing. Where it was reported, the variable effects of different abstinence periods clearly show that this is something that warrants further research.

It has been suggested that adolescents are more vulnerable to the cognitive deficits associated with alcohol consumption (Spear, 2018). Adolescence is a period where the brain undergoes rapid development, particularly in regions such as the prefrontal cortex which are important for mediating executive function and behavioral flexibility (Kim et al., 2017; Luikinga et al., 2018; Cullity et al., 2019). This state of change may lead to increased susceptibility to the negative effects of alcohol (Spear, 2018). It has even been suggested that within adolescence there are windows of increased vulnerability (Spear, 2015; Varlinskaya et al., 2020) and although this was beyond the scope of the research reviewed here, these could be teased out with further studies. In fact, there were very few studies that suggested that adolescents were more vulnerable, and indeed one study found that olfactory discrimination was more likely to be impaired in adults (Fernandez et al., 2017). However, most studies examined one age group only, and therefore it may be worth systematically probing age differences in future research.

While clearly important, there were very few studies in this review that looked at both male and female animals. In those that did, either both sexes were affected (West et al., 2018; Xing and Zou, 2018) or sex differences emerged suggesting males may be more prone to reversal errors (Varlinskaya et al., 2020), and females were more vulnerable to spatial learning deficits (Maynard et al., 2018). The latter study supports sex differences seen in human studies, where more severe alcohol-induced changes have often been observed in females (Wilhelm et al., 2015; McCaul et al., 2019). Although the biological basis for increased vulnerability in females is currently unknown, Maynard et al. (2018) suggest it may be due to lower basal rates of cell proliferation combined with higher apoptosis in females, making recovery from alcohol insult more difficult.

Interestingly, for the most part we did not uncover any major differences based on different administration method. One study did compare directly between i.p. and gavage administration, finding that only the former produced a deficit (Badanich et al., 2016). Generally, i.p. and gavage yield the same BAC at all time points (Chen et al., 2013). However, one study did observe a slightly increased BAC resulting from i.p. injection compared to gavage (Livy et al., 2003), where the difference could be attributed to metabolism by gastric alcohol dehydrogenase when alcohol was administered by gavage. This may account for the effect described above.

The intuitive observation that higher amounts of alcohol are more likely to cause a deficit may contribute to the seeming lack of consideration for the impact of other experimental protocols on study outcomes. Study designs focus on achieving highest possible intake levels, without accounting for other influential factors such as frequency of access, the amount of work required to obtain alcohol, and the impact of abstinence. Subject factors such as age, sex and genotype are also important considerations. This review highlights the need for further studies elucidating the effects of different alcohol administration methods on cognitive function.

We also propose that it would be worthwhile to consider the effects of a range of doses as well as individual differences in alcohol preference, rather than focusing only on the higher end. For example, in one study, improved cognition was reported following alcohol exposure in response to relatively lower intake levels (Varodayan et al., 2018). Indeed, where individual differences in drinking were taken into account, performance index could be stratified according to intake levels (Shnitko et al., 2019), however the lack of an alcohol naïve group in this study makes it harder to draw definitive conclusions regarding the effect of alcohol. A more comprehensive investigation of both dose-dependent deficits and individual differences in preference and vulnerability would be useful to help understand the mechanism of alcohol-induced change, as well as elucidating any potential protective effect of low alcohol intake.

Corollary to this, it is also worth noting that within the voluntary studies, sweetening appears to increase intake (Dwivedi et al., 2018; Pandey et al., 2020). High calorie sweeteners such as sugar and carbohydrates are thought to negatively impact cognition (Hawkins et al., 2018; Jacques et al., 2019; Sketriene et al., 2021), so it is important that control groups are calorie-matched (Charlton et al., 2019). In fact, this is relatively common practice in other forced models, such as Lieber DeCarli liquid diet. Furthermore, given that alcohol consumed by humans is typically sweetened in some form, any deleterious effects of sugar or calories on the human brain needs to be considered and recapitulated in our animal models.

Stress may also be contributing to cognitive deficits but as it is difficult to control for, its exact role is difficult to measure or assess. Clearly, non-voluntary methods of administration will pose more of a stress on the subject. The stress of the forced alcohol intake in addition may exacerbate the cognitive effects of alcohol (Tunstall et al., 2017; Hupalo et al., 2019a), especially in adolescents who are particularly vulnerable to the effects of both alcohol and stress (Spear, 2015, 2018; Romeo, 2017). In addition, both alcohol itself, as well as withdrawal effects of alcohol can act as acute stressors, leading to changes in corticotropin-releasing factor, and glucocorticoid dysregulation (Koob, 2008; Béracochéa et al., 2019; Hupalo et al., 2019b), which can in turn manifest as cognitive deficits. With many behavioral tests being performed while animals are in withdrawal, this could be impacting cognition. However, this is not often accounted for in studies, and in fact many studies failed to report the length of abstinence and/or signs of withdrawal where abstinence periods were short. In addition, inherently stressful behavioral tests, such as the Morris water maze and Barnes maze, may compound this withdrawal stress, further impacting the animal’s performance. It’s worth noting too that voluntary methods of administration did not result in increased anxiety in animals in the way that many non-voluntary methods did (Dwivedi et al., 2018; Jacques et al., 2019; Pandey et al., 2020). To determine the cognitive effects of alcohol, we must account for and assess the role of stress, which would also commonly occur in the human condition, but also look to those deficits that persist outside it.

In summary, from the collective literature, a chronic voluntary model that embraces variability and stratifies individuals into groups based on alcohol consumption (with heavy drinkers reaching 6 g/kg), which can be assisted by sweetening, appears to produce deficits with minimal stress confounds. Withdrawal and abstinence periods also play a significant role, and this should be explored and reported more accurately in the future. In addition, behavioral tests that are not inherently stressful or reliant on aversive stimuli would also reduce the confound of stress, giving clearer results on the effect of alcohol. A battery of such tests would help ascertain nuances of the cognitive functions impacted. This research should be conducted in both females and males and compared across age groups. In addition, due to the high variability in methods, it would be useful to have some consistent reporting guidelines that include blood alcohol concentration and abstinence period to form a more holistic understanding of the effects of alcohol intake on cognition.

## Author Contributions

Both authors listed have made a substantial, direct, and intellectual contribution to the work, and approved it for publication.

## Conflict of Interest

The authors declare that the research was conducted in the absence of any commercial or financial relationships that could be construed as a potential conflict of interest.

## Publisher’s Note

All claims expressed in this article are solely those of the authors and do not necessarily represent those of their affiliated organizations, or those of the publisher, the editors and the reviewers. Any product that may be evaluated in this article, or claim that may be made by its manufacturer, is not guaranteed or endorsed by the publisher.
